# Adaptive Communication Model for QoS in Vehicular IoT Systems Using CTMC

**DOI:** 10.3390/s25061818

**Published:** 2025-03-14

**Authors:** Adeel Iqbal, Tahir Khurshaid, Ali Nauman, Sung Won Kim

**Affiliations:** 1 School of Computer Science and Engineering, Yeungnam University, Gyeongsan-si 38541, Republic of Korea; adeeliqbal@yu.ac.kr (A.I.); swon@yu.ac.kr (S.W.K.); 2 Department of Electrical Engineering, Yeungnam University, Gyeongsan-si 38541, Republic of Korea; tahir@ynu.ac.kr

**Keywords:** V-IoT, CTMC, AMSA, C-ITS, vehicular networks, throughput, QoS

## Abstract

Vehicular Internet of Things (V-IoT) systems will be critical in advancing intelligent transportation networks because of the easy communication they make possible between vehicles, roadside infrastructure, and other network entities. Integrating adaptive IoT-based communication models will increase resource utilization and allow multiple communications in vehicular networks. This work proposes an Adaptive Multi-mode Spectrum Access (AMSA) approach for optimal Quality of Service (QoS) in multi-class V-IoT networks. Unlike traditional static spectrum access methods, AMSA switches between interweave, underlay, and coexistence modes based on network conditions. Our results indicate that AMSA improves spectrum usage by 56% over static spectrum access improves throughput by 110%, and reduces delay for low-priority traffic by up to 47.5%. This new integration offers robust vehicular communication with optimal resource allocation under different network scenarios.

## 1. Introduction

The advent of Fifth Generation (5G) technology has revolutionized the telecommunications landscape, enabling unprecedented data rates, ultra-reliable low-latency communication, and massive device connectivity. However, as the demand for more sophisticated applications, such as autonomous vehicles, smart cities, and extended reality, continues to grow, the limitations of 5G are becoming apparent [1]. Emerging applications require even lower latency, higher capacity, and more robust connectivity than what 5G can offer. Consequently, researchers and industry stakeholders are already exploring the next generation of wireless communication: the Sixth Generation (6G) cellular network. With promises of terahertz spectrum utilization, AI-driven networks, and ubiquitous connectivity, 6G aims to address these challenges and enable a fully connected, intelligent ecosystem [2].

In the evolving landscape of intelligent transportation networks, Vehicular Internet of Things (V-IoT) systems play a crucial role by enabling seamless communication between vehicles, roadside infrastructure, and other entities within the network [3]. For example, V-IoT systems have been successfully implemented in smart city projects, such as Singapore’s real-time traffic management system, where connected vehicles interact with intelligent traffic lights to help reduce congestion [4,5]. Additionally, in Europe, pilot programs like the Cooperative Intelligent Transport Systems (C-ITS) [6,7] have demonstrated the potential of V-IoT to enhance road safety through vehicle-to-vehicle (V2V) and vehicle-to-infrastructure (V2I) communication, which aids in collision avoidance.

Despite advancements in V-IoT systems, significant challenges remain in meeting the Quality of Service (QoS) requirements for various vehicular applications. High-priority applications, such as collision avoidance, necessitate ultra-low latency and reliable communication, while low-priority, delay-tolerant applications like telemetry data transmission require efficient resource utilization without excessive packet loss. The dynamic and resource-constrained nature of vehicular networks demands adaptive communication models that can effectively balance these competing needs.

Several research studies have explored spectrum-sharing protocols within V-IoT; however, these protocols often lack scalability in dynamic vehicular environments. For instance, Lee [8] proposed a reservation-based MAC protocol that successfully reduced delay but resulted in more frequent spectrum handoffs, which affected service continuity. Similarly, Shakeel et al. [9] expanded on Continuous-Time Markov Chains (CTMC) to develop hybrid interweave-underlay systems; however, they did not incorporate waiting mechanisms, leading to increased drop rates for non-real-time traffic. These limitations underscore the need for a more adaptable spectrum access strategy that optimally balances QoS requirements.

This work addressed these challenges and proposed a novel solution. The details are as follows:

- A novel Adaptive Multi-mode Spectrum Access (AMSA) model integrating interweave, underlay, and coexistence spectrum access modes.
- A CTMC framework for analyzing AMSA’s impact on QoS in multi-class V-IoT networks.
- The introduction of a waiting mechanism to reduce drop rates for low-priority traffic, enhancing spectrum utilization.
- A comprehensive performance evaluation demonstrating up to 56% improvement in spectrum utilization and a 110% increase in throughput over conventional methods.

## 2. Literature Survey

The growing demand for efficient vehicular communication systems has motivated numerous studies aiming at improving the QoS for V-IoT networks. In this section, we review the research contributions of these studies to identify gaps in the current state of the art and highlight the relevance of our proposed solution.

Singh and Khatri (2024) [10] have discussed spectrum-sharing approaches in Agriculture IoT networks, highlighting the need for an efficient communication protocol in agriculture IoT systems. Their work examines the use of fixed and dynamic channel allocation and performs an analysis using the non-priority algorithm, and two offshoots of the reserve channel algorithm, namely, “no permanent channel” and “with permanent channel”. The work focuses on the improvement of the system in terms of call blocking, forced termination of calls, and handover failure. The proposed study did not consider throughput, and delay. Wu et al. (2023) [11] have proposed the SpectrumChain, a blockchain-based framework focusing on dynamic spectrum-sharing strategies for next-generation networks. While their proposed framework was a further advance in dynamic spectrum access, their model was more oriented toward improving the security of the system by incorporating blockchain technology, without catering to the specialized needs of V-IoT networks.

Okegbile and Maharaj (2021) [12] evaluated the performance of a hybrid spectrum access model in cognitive radio networks that switch dynamically between underlay and overlay modes based on channel sensing. The model optimizes the throughput while minimizing interference with the primary users. Using stochastic geometry, the success probability, throughput, and age of information are analyzed, proving that the hybrid approach outperforms standalone underlay or overlay schemes while being very close to real-world modeling. Ferdouse et al. (2019) [13] focused on the problem of resource allocation in Cloud-Radio Access Networks (C-RANs), addressing two challenges in dense networks: interference and scalability. Their approach formulates optimization problems for communication resource blocks, power, and computing resources, and baseband unit remote radio head mapping. It involves a double-sided auction-based approach for minimizing the response time while improving bandwidth utilization, signal quality, and user capacity in 5G C-RANs.

In [8], the author proposes an efficient reservation-based Medium Access Control (MAC) protocol featuring effective delay reduction of multi-priority traffic over slotted multi-channel distributed cognitive radio networks. While efficient, such an approach often leads to spectrum handoffs. Therefore, these would result in service interruptions and increased drop rate for low-priority users. The aforesaid necessitates low-disruption handoff mechanisms with effective use of the spectrum. Shakeel et al. (2019) [9] extend CTMC to hybrid interweave-underlay systems. Their approach is limited to cognitive users and Wifi networks and does not consider waiting mechanisms for low-priority 5G traffic, though it considers dynamic spectrum access. This results in higher drop rates for non-real-time applications. Iqbal et al. (2022) [14] proposed an enhanced hybrid spectrum access model that combines interweave and underlay modes. The method proposed by them improves the spectrum utilization but is also limited to Wifi networks for cognitive D2D traffic for low-proximity users to reduce drop rates for delay-tolerant traffic. The model also does not consider 5G networks with vehicular traffic.

The literature reviewed contributes much to spectrum access and QoS provisioning for various systems. However, a number of key gaps still exist, such as limited adaptability, since the existing models often fail to address the dynamic nature of vehicular networks, especially for multi-class traffic. Similarly, the coexistence states are lacking because most of the studies overlook the coexistence modes that may enhance the spectrum efficiency and reduce the drop rates for delay-tolerant traffic. Finally, the QoS provisioning for high-priority applications of V-IoT traffic is not considered in the existing literature, undermining their reliability in critical scenarios. This paper proposes the AMSA model, which combines dynamic spectrum access schemes and a CTMC framework for the purpose of optimizing the throughput, delay, and spectrum efficiency of multi-class V-IoT systems. Coexistence and waiting states in the AMSA model can meet the demand for a well-balanced QoS for both delay-sensitive and delay-tolerant applications—a deficiency in other works. It means that the approach proposed here fits the demand for modern V-IoT networks, with challenges resulting from high mobility and diverse traffic, by being strong and efficient.

## 3. System Model

**Figure 1:**
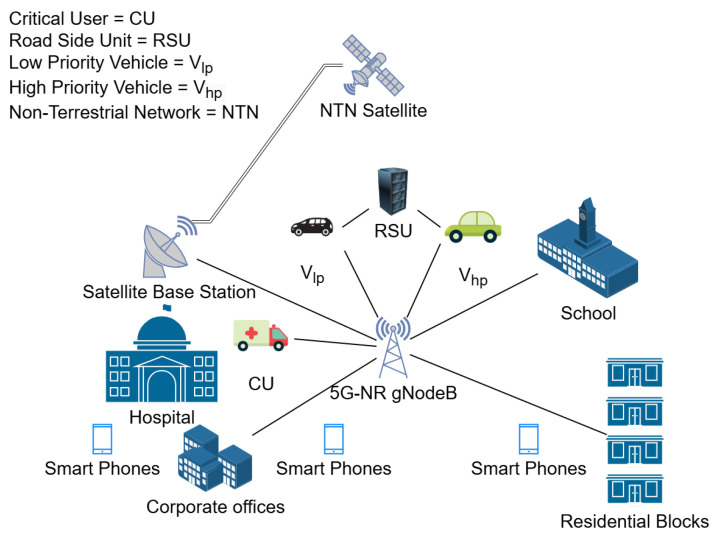
System model: V-IoT system in a smart city environment.

In this work, we consider a V-IoT system where vehicles communicate with roadside infrastructure and other vehicles through both direct and infrastructure-assisted communication. The system operates under a hybrid interweave-underlay spectrum access scheme, ensuring efficient utilization of resources while maintaining QoS for multi-class vehicular applications. The system is categorized into three types of users based on their application requirements. Critical Users (CUs) are the legitimate users with the highest priority to access the spectrum, then comes the High-Priority Vehicles ($V_{\mathrm{hp}}$) handling delay-sensitive traffic, such as collision avoidance and emergency messages, and Low-Priority Vehicles ($V_{\mathrm{lp}}$) managing delay-tolerant traffic, such as infotainment and telemetry data. Figure 1 illustrates the pictorial representation of the system model.

The arrival of CUs follows an independent Poisson process, with exponentially distributed inter-arrival times [9,15]. A Preemptive Resume Priority (PRP) queuing mechanism [16,17] is employed to prioritize spectrum access, ensuring that CU traffic can preempt $V_{\mathrm{hp}}$ and $V_{\mathrm{lp}}$ traffic. Similarly, $V_{\mathrm{hp}}$ traffic preempts $V_{\mathrm{lp}}$ traffic when necessary. This mechanism also allows $V_{\mathrm{lp}}$ traffic to wait rather than being dropped, enhancing overall spectrum utilization. The hybrid spectrum access model [18,19,20] leverages interweave and underlay techniques. When a vehicle detects an idle spectrum channel, it utilizes interweave access to achieve high data rates without interfering with CUs. Underlay spectrum access is employed when the CU occupies the spectrum. In underlay mode, vehicles adjust their transmission power below an interference threshold to avoid causing interference to CU traffic. The data rate in the case of interweave spectrum access is given by the following equation:

(1) $$\zeta =W\log_{2}\left(1+\frac{p_{\alpha }g_{\alpha }}{n}\right),$$

where *W* represents the communication bandwidth, $p_{\alpha }$ represents the transmission power of vehicle α, $g_{\alpha }$ is the channel gain, and *n* is the power of Additive White Gaussian Noise (AWGN). Equation (1) represents the achievable data rate under interweave access, where vehicles opportunistically access the spectrum only when it is idle. This approach maximizes spectrum utilization without interfering with ongoing CU transmissions.

The data rate $\zeta_{u}^{\alpha }$ in the case of given underlay access is constrained by the interference threshold and is given by the following equation:

(2) $$\zeta_{u}^{\alpha }=W\log_{2}\left(1+\frac{p_{\alpha }g_{\alpha }}{n+p_{cu}g_{cu\alpha }}\right),$$

where $p_{cu}$ represents the transmission power of the primary user, and $g_{cu\alpha }$ represents the channel gain between the CU and vehicle α. Equation (2) accounts for the presence of CU transmissions by incorporating the interference power term $p_{cu}g_{cu\alpha }$ in the denominator. This ensures that underlay users adjust their power levels accordingly to maintain reliable communication without exceeding the interference threshold imposed by the CU. To improve energy efficiency, a non-switching spectrum handoff policy is adopted. Vehicles maintain their assigned channels and resume transmission once the CUs vacate the spectrum. The channel state is monitored using a binary hypothesis model:

(3) $$x\left(t\right)=\left\{\begin{array}{cc} n(t), & I\\ b(t)+n(t), & B \end{array}\right.$$

where *I* represents an idle state, *B* represents a busy state, n(t) is the AWGN, and b(t) is the transmitted signal of the vehicle. Ideal spectrum sensing is assumed to avoid errors in detection. The proposed system model ensures efficient spectrum utilization and QoS for multi-class vehicular applications, making it a robust solution for dynamic V-IoT environments.

### Proposed AMSA Algorithm

The Algorithm 1 is proposed to implement the improved version of the hybrid spectrum access mechanism. The AMSA algorithm ensures that CU gets the spectrum without any interruption, and the $V_{\mathrm{hp}}$ and $V_{\mathrm{lp}}$ are allocated spectrum efficiently while maintaining QoS.
**Algorithm 1** AMSA Spectrum Allocation Algorithm**Input:** Spectrum state *S*, Critical User (CU), High-Priority Vehicles ($V_{hp}$), Low-Priority Vehicles ($V_{lp}$), Channel Utilization Strategy (IW = Interweave, HB = Hybrid Interweave-Underlay, WT = Waiting)**Output:** Efficient spectrum allocation maintaining QoSInitialize: Vehicles scan for available channels**if **S= Idle **then**   Tx = IW**end if****if **$V_{hp}$ preempts $V_{lp}$ **then**    $V_{lp}$ switches HB**end if****if** CU arrives **then**   **if** Channel is Idle **then**       CU occupies the channel immediately   **else**       Preempt current user ($V_{hp}$ or $V_{lp}$) and vacate the channel for CU   **end if****end if****if **$V_{lp}$ preempted by $V_{hp}$ **then**    Move $V_{lp}$ to WT**end if****while** Channel becomes available **do**     Resume transmission**end while**Terminate session upon successful data transmission

## 4. Steady-State Performance Evaluation for AMSA Model

This section presents the steady-state analysis of the AMSA system for V-IoT networks. Utilizing CTMC modeling, we evaluate the system’s performance under various network conditions and traffic intensities to ensure QoS for multi-class vehicular traffic.

The analysis focuses on multiple configurations:

1. Baseline Network with Critical User Traffic (CU-VT): A network where critical vehicular traffic accesses the spectrum using traditional techniques without adaptive modes.
2. Single-Class Vehicular Traffic with Interweave Access (CU-VT-IW): A system where vehicular traffic opportunistically accesses idle spectrum, prioritizing spectrum efficiency.
3. Multi-Class Vehicular Traffic with Hybrid Interweave-Underlay Spectrum Access (CU-VT-HB): A model incorporating interweave and underlay modes to handle both high-priority and low-priority traffic.
4. Multi-Class Vehicular Traffic with AMSA (CU-VT-AMSA): The proposed AMSA model integrates coexistence and waiting mechanisms to optimize spectrum utilization and quality of service (QoS). In CU-VT-HB, under higher loads, low-priority traffic is dropped from the system due to resource unavailability. In AMSA, we allocate resources to low-priority users to allow them to continue using the same spectrum while in a waiting state, avoiding the need for spectrum handoff and rejoining the system for new resources. Thus, in CU-VT-AMSA, low-priority users are compensated through a coexistence mechanism.

The network state is represented as $S=\{I,C,V_{hp},V_{lp},CV_{hp},CV_{lp},V_{hp}V_{lp}\}$, where *I* represents the Idle state with no active users. *C* represents the CU occupying the spectrum. $V_{hp}$ and $V_{lp}$ represent the high-priority and low-priority vehicular users utilizing spectrum in interweave mode. The $CV_{hp}$ is the Coexistence of critical and high-priority vehicular users using hybrid access. The $CV_{lp}$ is the Coexistence of critical and low-priority vehicular users using hybrid access and the $V_{hp}V_{lp}$ is the Coexistence of high-priority and low-priority vehicular users using hybrid access.

Transitions between states occur due to the Arrivals of the high-priority $\left(\lambda_{hp}\right)$, low-priority $\left(\lambda_{lp}\right)$, and CU $\left(\lambda_{c}\right)$. The Departures shows the high-priority $\left(\mu_{hp}\right)$, low-priority $\left(\mu_{lp}\right)$, and critical user $\left(\mu_{c}\right)$. The Spectrum Handoffs are triggered by preemptive access from higher-priority users or channel availability. The steady-state probabilities π are computed by solving the global balance equations derived from the CTMC transition rate matrix *Q*. These probabilities quantify the likelihood of the system being in any given state.

(4) $$\sum_{\pi \in S}\pi \left(\beta \right)=1$$

The probabilities are used to calculate the performance metrics. The Throughput is the weighted sum of data rates for $V_{hp}$ and $V_{lp}$. The Spectrum Efficiency is the Throughput per unit bandwidth and the Average Delay is the Time spent by $V_{lp}$ traffic in the waiting state. The Comparative Configurations are performed in the next sections to show the validity of the analysis.

### 4.1. Critical Users with Vehicular Traffic (CU-VT)

**Figure 2:**
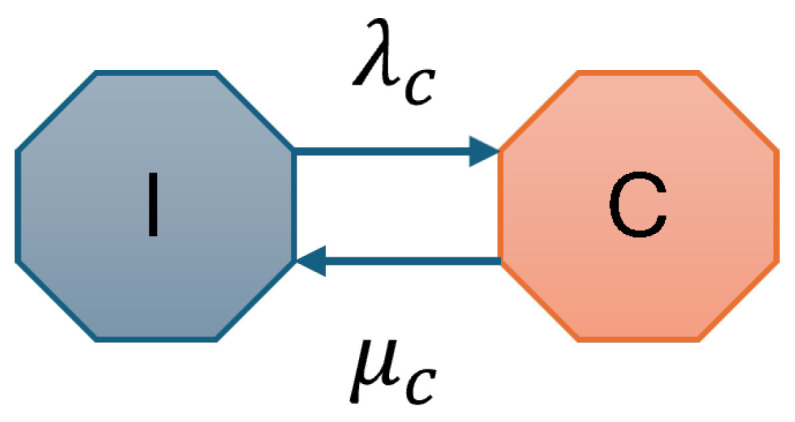
State transition diagram for CU-VT.

This configuration serves as the baseline for evaluating the performance of the spectrum access mechanisms in vehicular networks. The CU-VT considers only the CU and Figure 2 depicts the state transition diagram for this scenario. The CTMC model includes two primary states: *I* (Idle) and *C* occupied by CU. The arrival and departure of CU traffic govern transitions between these states.

The arrival and departure of traffic in the network are modeled as a Poisson process with exponentially distributed arrival and departure rates $\lambda_{c}$ and $\mu_{c}$, respectively. The system starts in state *I*, indicating the channel is idle. When CU traffic arrives, the system transitions to state *C* with rate $\lambda_{c}$. After completing its session, the CU departs, and the system transitions back to state *I* with rate $\mu_{c}$. The CTMC for CU-VT is defined as S={I,C}, where *I* is the Idle state with no active users and *C* shows the state occupied by CU. To represent transitions among the states of *S*, the state transition matrix Q is given as follows:

(5) $$Q=\begin{array}{cc} & \begin{array}{cc} I\;\; & \;\;C \end{array}\\ \begin{array}{c} I\\ C \end{array} & \left[\begin{array}{cc} -\lambda_{c} & \mu_{c}\\ \lambda_{c} & -\mu_{c} \end{array}\right] \end{array}$$

For the CU-VT scenario, the flow balance equations [9,21] ensure that the rate of transitions into and out of each state is equal. These equations are given as follows:

(6) $$\pi_{I}\lambda_{c}=\pi_{C}\mu_{c},$$

where $\pi_{I}$ and $\pi_{C}$ are the steady-state probabilities for the states *I* and *C*, respectively, where the normalization condition is:

(7) $$\pi_{I}+\pi_{C}=1,$$

Solving (6) and (7), we obtain:

(8) $$\pi_{I}=\frac{\mu_{c}}{\lambda_{c}+\mu_{c}},$$

(9) $$\pi_{C}=\frac{\lambda_{c}}{\lambda_{c}+\mu_{c}}.$$

**Figure 3:**
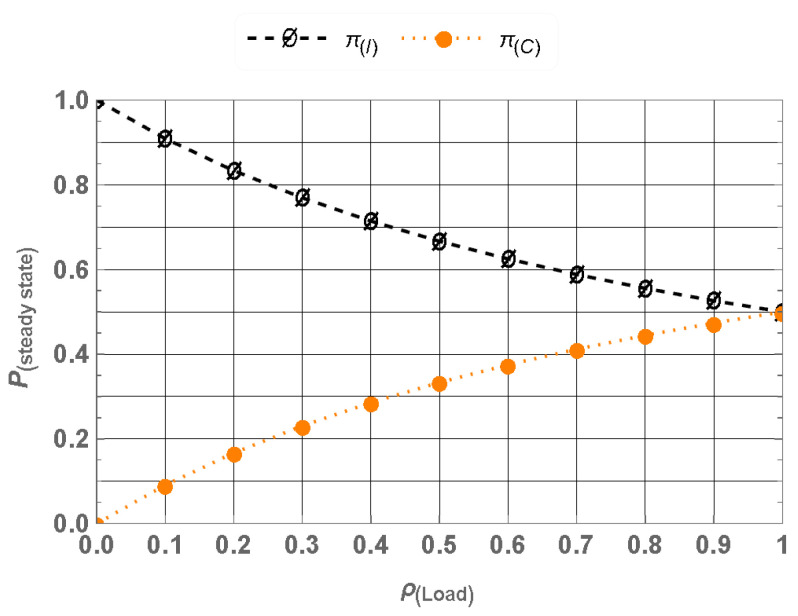
Steady-state probabilities for CU-VT.

To evaluate the performance using the steady-state probabilities π(β), where β∈S represents the $\rho_{Load}$, we compute the spectrum utilization, which is given by $\pi_{C}$, representing the proportion of time the spectrum is occupied, and the Idle Probability that is represented by $\pi_{I}$, indicating the proportion of time the spectrum is idle and available for other users. The steady-state probabilities $\pi_{I}$ and $\pi_{C}$ for varying traffic loads $\rho =\frac{\lambda_{c}}{\mu_{c}}$ are shown in Figure 3. The results highlight that even under high traffic loads, there is a potential for opportunistic spectrum usage by other users. This emphasizes the importance of advanced spectrum access mechanisms to fully utilize the available spectrum resources.

### 4.2. Single-Class Vehicular Traffic with Interweave Access (CU-VT-IW)

**Figure 4:**
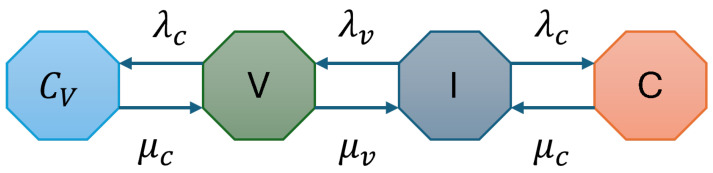
State transition diagram for CU-VT-IW.

In the second configuration, we introduce interweave spectrum access to improve spectrum utilization in vehicular networks. This scenario considers CUs with critical vehicular traffic following the interweave spectrum access. The Vehicular Users (VTs) exploit the idle spectrum opportunistically, ensuring minimal interference with CUs. Figure 4 depicts the state transition diagram for this scenario.

The traffic arrivals for both CUs and VTs are modeled as independent Poisson processes with arrival rates $\lambda_{c}$ and $\lambda_{v}$, respectively. The service times are exponentially distributed with departure rates $\mu_{c}$ for CUs and $\mu_{v}$ for VTs. The CTMC model includes four states: *I* (Idle), *C* (Occupied by CU), *V* (Occupied by VTs), and $C_{v}$ (VT waiting state while preempting CUs). Figure 4 depicts the transitions among these states.

The CTMC for CU-VT-IW is defined as $S=\{I,C,V,C_{v}\}$, and the transition rate matrix Q is given as follows:

(10) $$Q=\begin{array}{cc} & \begin{array}{cccc} \;\;I & \;\;C & \;\;\;\;\;\;\;V & \;\;\;C_{v} \end{array}\\ \begin{array}{c} I\\ C\\ V\\ C_{v} \end{array} & \left[\begin{array}{rrrr} -(\lambda_{c}+\lambda_{v}) & \lambda_{c} & \lambda_{v} & 0\\ \mu_{c} & -\mu_{c} & 0 & 0\\ \mu_{v} & 0 & -(\mu_{v}+\lambda_{c}) & \lambda_{c}\\ 0 & 0 & \mu_{c} & -\mu_{c} \end{array}\right] \end{array}$$

The flow balance equations for CU-VT-IW ensure that the rates of transitions into and out of each state are balanced. These equations are given as follows:

(11) $$\begin{array}{cc} \pi_{I}\left(\lambda_{c}+\lambda_{v}\right) & =\pi_{C}\mu_{c}+\pi_{V}\mu_{v},\\ \pi_{C}\mu_{c} & =\pi_{I}\lambda_{c},\\ \pi_{V}\left(\lambda_{c}+\mu_{v}\right) & =\pi_{I}\lambda_{v}+\pi_{C_{v}}\mu_{c},\\ \pi_{C_{v}}\mu_{c} & =\pi_{V}\lambda_{c}. \end{array}$$

Solving these equations along with the normalization condition:

(12) $$\pi_{I}+\pi_{C}+\pi_{V}+\pi_{C_{v}}=1,$$

we obtain the steady-state probabilities $\pi_{I},\pi_{C},\pi_{V},\mathrm{and}\pi_{C_{v}}$:

(13) $$\begin{array}{cc} \pi_{I} & =\frac{\mu_{c}\mu_{v}}{\left(\lambda_{c}+\mu_{c}\right)\left(\lambda_{v}+\mu_{v}\right)},\\ \pi_{C} & =\frac{\lambda_{c}\mu_{v}}{\left(\lambda_{c}+\mu_{c}\right)\left(\lambda_{v}+\mu_{v}\right)},\\ \pi_{V} & =\frac{\lambda_{v}\mu_{c}}{\left(\lambda_{c}+\mu_{c}\right)\left(\lambda_{v}+\mu_{v}\right)},\\ \pi_{C_{v}} & =\frac{\lambda_{c}\lambda_{v}}{\left(\lambda_{c}+\mu_{c}\right)\left(\lambda_{v}+\mu_{v}\right)}. \end{array}$$

**Figure 5:**
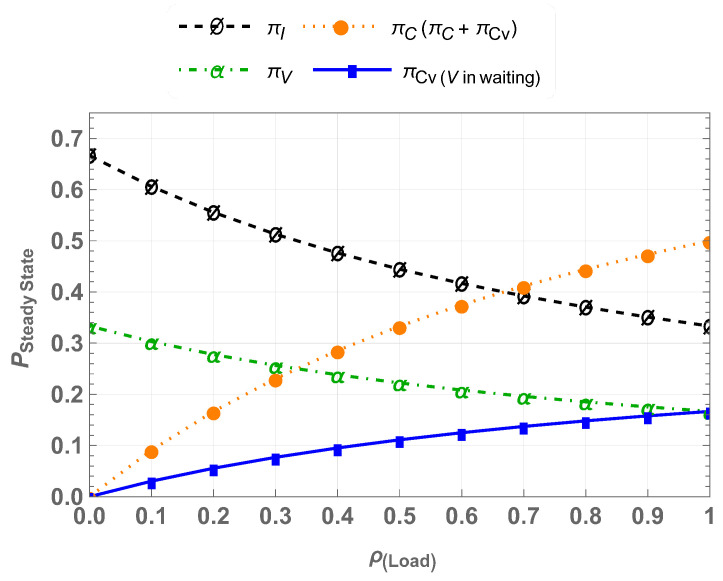
Steady -state probabilities for CU-VT-IW.

For varying traffic loads $\rho =\frac{\lambda_{c}}{\mu_{c}}$, the steady-state probabilities of CU-VT-IW are shown in Figure 5. The results indicate the spectrum utilization improves as $\pi_{C}$ and $\pi_{C_{v}}$ increase with higher CU loads, while $\pi_{V}$ decreases, showing reduced opportunities for vehicular users to access the spectrum under heavy CU traffic. This emphasizes the importance of coexistence mechanisms to improve spectrum utilization without compromising CU performance.

### 4.3. Multi-Class Vehicular Traffic with Hybrid Spectrum Access (CU-VT-HB)

**Figure 6:**
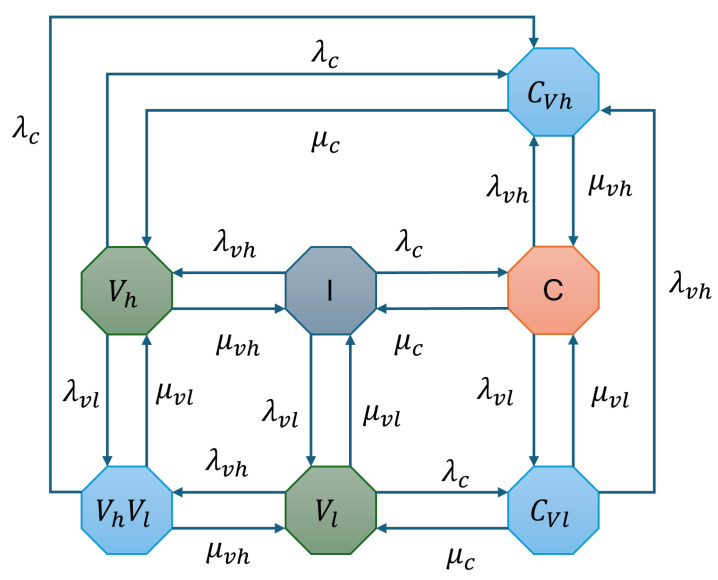
State transition diagram for CU-VT-HB.

This configuration incorporates interweave and underlay modes to effectively accommodate multi-class vehicular traffic. The CU-VT-HB scenario prioritizes the $V_{hp}$ while allowing $V_{lp}$ to utilize idle or shared spectrum resources. The coexistence mechanisms enable improved resource management, but there is a drop rate for $V_{lp}$ in this configuration when the system is in a $V_{h}V_{l}$ state and there is an arrival of CU with $\lambda_{c}$ when the system is moved to a $C_{Vh}$ state and the $V_{l}$ is dropped from the system; similarly when the system is in a $C_{Vl}$ state and there is an arrival of $VT_{hp}$, the system is moved to a $C_{Vh}$ state. These scenarios only occur at higher system loads and Figure 6 illustrates the state transition diagram for this configuration.

The system incorporates CU, $VT_{hp}$, and $VT_{lp}$, with their arrivals modeled as independent Poisson processes. The respective arrival rates are denoted as $\lambda_{c}$, $\lambda_{vh}$, and $\lambda_{vl}$ for CU, $VT_{hp}$, and $VT_{lp}$, while their corresponding departure rates are $\mu_{c}$, $\mu_{vh}$, and $\mu_{vl}$.

The CTMC representation consists of several states: *I* (Idle state where the channel is unoccupied), *C* (Channel occupied by CU), $V_{h}$ (Channel occupied by $VT_{hp}$), and $V_{l}$ (Channel occupied by $VT_{lp}$). Additionally, preemption states include $C_{vh}$, where CU takes over the channel from $VT_{hp}$, and $C_{vl}$, where CU preempts $VT_{lp}$, potentially causing a drop in $V_{l}$ if a high-priority vehicle arrives. The state $V_{hp}V_{lp}$ represents the coexistence of high- and low-priority vehicles, but if a CU arrives, $VT_{lp}$ is forced to drop.

Figure 6 illustrates the transitions between these states. The CTMC for CU-VT-HB is formally defined as $S=\{I,C,V_{hp},V_{lp},C_{vh},C_{vl},V_{hp}V_{lp}\}$, with the transition rate matrix Q provided in Equation (14).

(14) $$Q=\begin{array}{cc} & \begin{array}{lllllll} \;I & \;C & \;V_{h} & \;V_{l} & \;C_{\mathrm{vh}} & \;C_{\mathrm{vl}} & \;V_{h}V_{l} \end{array}\\ \begin{array}{c} I\\ C\\ V_{h}\\ V_{l}\\ C_{\mathrm{vh}}\\ C_{\mathrm{vl}}\\ V_{h}V_{l} \end{array} & \left[\begin{array}{ccccccc} -(\lambda_{c}+\lambda_{vh}+\lambda_{vl}) & \lambda_{c} & \lambda_{vh} & \lambda_{vl} & 0 & 0 & 0\\ \mu_{c} & -(\mu_{c}+\lambda_{vh}+\lambda_{vl}) & 0 & 0 & \lambda_{vh} & \lambda_{vl} & 0\\ \mu_{vh} & 0 & -(\mu_{vh}+\lambda_{c}+\lambda_{vl}) & 0 & \lambda_{c} & 0 & \lambda_{vl}\\ \mu_{vl} & 0 & 0 & -(\mu_{vl}+\lambda_{c}+\lambda_{vl}) & 0 & \lambda_{c} & \lambda_{vh}\\ 0 & \mu_{vh} & \mu_{c} & 0 & -(\mu_{c}+\mu_{vh}) & 0 & 0\\ 0 & \mu_{vl} & 0 & \mu_{c} & 0 & -(\mu_{c}+\mu_{vl}) & 0\\ 0 & 0 & \mu_{vl} & \mu_{vh} & \lambda_{c} & 0 & -(\mu_{vh}+\mu_{vl}+\lambda_{c}) \end{array}\right] \end{array}$$

The flow balance equations for CU-VT-HB ensure that the rates of transitions into and out of each state are balanced. These equations are given as follows:

(15) $$\begin{array}{cc} \pi_{I}\left(\lambda_{c}+\lambda_{vh}+\lambda_{vl}\right) & =\pi_{C}\mu_{c}+\pi_{V_{h}}\mu_{vh}+\pi_{V_{l}}\mu_{vl},\\ \pi_{C}\left(\mu_{c}+\lambda_{vh}+\lambda_{vl}\right) & =\pi_{I}\lambda_{c}+\pi_{C_{vh}}\mu_{vh}+\pi_{C_{vl}}\mu_{vl},\\ \pi_{V_{h}}\left(\mu_{vh}+\lambda_{c}+\lambda_{vl}\right) & =\pi_{I}\lambda_{vh}+\pi_{V_{h}V_{l}}\mu_{vl}+\pi_{C_{vh}\mu_{c}},\\ \pi_{V_{l}}\left(\mu_{vl}+\lambda_{vh}+\lambda_{c}\right) & =\pi_{I}\lambda_{vl}+\pi_{C_{vl}}\mu_{c}+\pi_{V_{h}V_{l}}\mu_{vh},\\ \pi_{C_{vh}}\left(\mu_{vh}+\mu_{c}\right) & =\pi_{V_{h}}\lambda_{c}+\pi_{C}\lambda_{vh}+\pi_{C_{vl}}\lambda_{vh}+\pi_{V_{vh}V_{vl}}\lambda_{c},\\ \pi_{C_{vl}}\left(\mu_{c}+\mu_{vl}+\lambda_{vh}\right) & =\pi_{V_{l}}\lambda_{c}+\pi_{C}\lambda_{vl},\\ \pi_{V_{h}V_{l}}\left(\mu_{vh}+\mu_{vl}+\lambda_{c}\right) & =\pi_{V_{h}}\lambda_{vl}+\pi_{V_{h}}\lambda_{vl}. \end{array}$$

**Figure 7:**
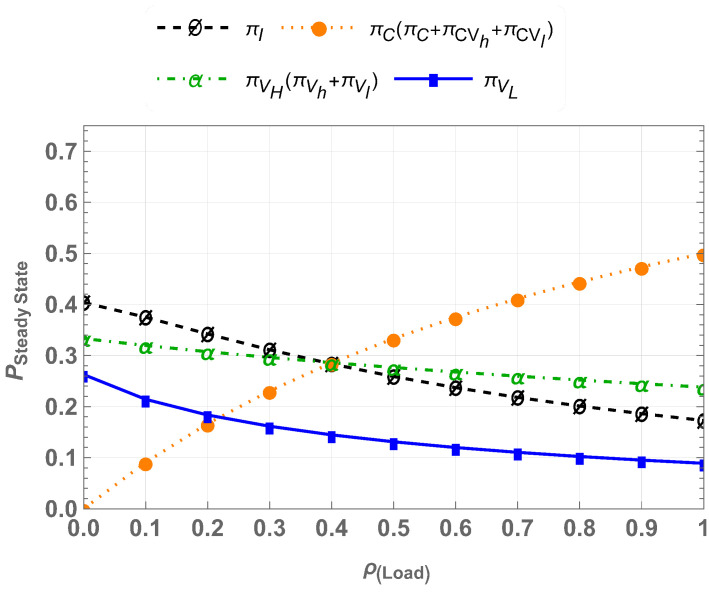
Steady-state probabilities for CU-VT-HB.

Solving these equations along with the normalization condition:

(16) $$\pi_{I}+\pi_{C}+\pi_{V_{h}}+\pi_{V_{l}}+\pi_{C_{vh}}+\pi_{C_{vl}}+\pi_{V_{h}V_{l}}=1,$$

we obtain the steady-state probabilities $\pi_{I},\pi_{C},\pi_{V_{h}},\pi_{V_{l}},\pi_{C_{vh}},\pi_{C_{vl}},\mathrm{and}\pi_{V_{h}V_{l}}$. For varying traffic loads $\rho =\frac{\lambda_{c}}{\mu_{c}}$, the steady-state probabilities of CU-VT-HB are shown in Figure 7. The results indicate that the hybrid access mechanism accommodates multi-class traffic effectively. The coexistence of high-priority and low-priority traffic improves spectrum utilization, while there is still a drop rate for $V_{lp}$ users at higher traffic load.

### 4.4. Multi-Class Vehicular Traffic with AMSA (CU-VT-AMSA)

**Figure 8:**
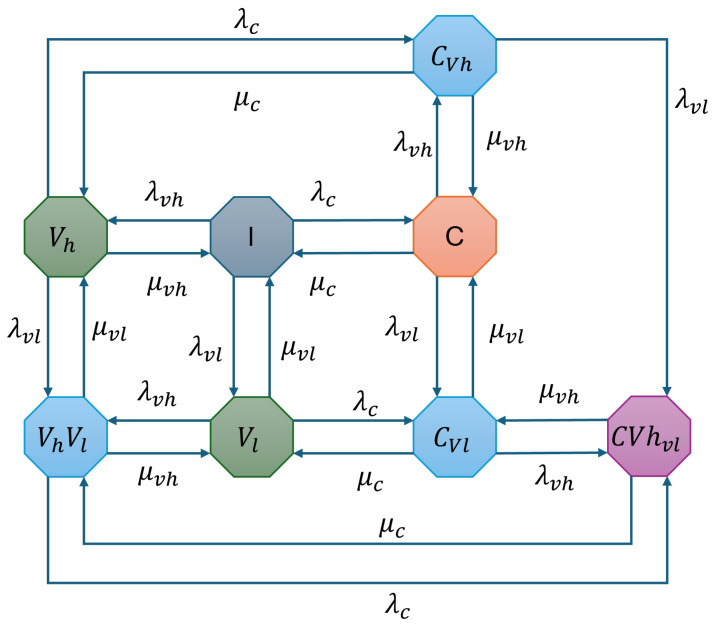
State transition diagram for CU-VT-AMSA.

The proposed AMSA model enhances spectrum utilization and QoS provisioning by overcoming the limitations of traditional and hybrid access mechanisms. The CU-VT-AMSA integrates coexistence and waiting mechanisms to improve system performance. In the CU-VT-HB configuration, the $V_{lp}$ is either not allowed to enter the system or dropped from the system at high load. To overcome this limitation, we utilized the coexistence mode, where $V_{lp}$ is authorized to wait for their turn instead of being dropped from the system. Figure 8 illustrates the state transition diagram for this advanced configuration.

- Coexistence Mechanisms: Enable simultaneous spectrum usage by $V_{hp}$ and $V_{lp}$, enhancing spectrum utilization.
- Waiting States: Reduce drop rates for $V_{lp}$ traffic by providing opportunities to reaccess the spectrum.
- Prioritization of $V_{hp}$: Ensures QoS for delay-sensitive applications.

The system supports CU, $VT_{hp}$, and $VT_{lp}$ with arrivals modeled as independent Poisson processes. The arrival and departure rates are the same as in previous configurations. The rest of the CTMC states are also the same; there is only one addition in the state space diagram, which is the $CVh_{vl}$ state that enables CU in interweave and $VT_{hp}$ in underlay, and $VT_{lp}$ in the waiting state, enabling simultaneous utilization of the spectrum by all three.

The CTMC for CU-VT-AMSA is defined as $S=\{I,C,V_{h},V_{l},C_{Vh},C_{Vl},V_{h}V_{l},CVh_{Vl}\}$, and the transition rate matrix Q is illustrated in Equation (17).

(17) $$Q=\begin{array}{cc} & \begin{array}{llllllll} \;I & \;C & \;V_{h} & \;V_{l} & \;C_{Vh} & \;C_{Vl} & \;V_{h}V_{l} & \;CVh_{Vl} \end{array}\\ \begin{array}{c} I\\ C\\ V_{h}\\ V_{l}\\ C_{Vh}\\ C_{Vl}\\ V_{h}V_{l}\\ CVh_{Vl} \end{array} & \left[\begin{array}{cccccccc} -(\lambda_{c}+\lambda_{vh}+\lambda_{vl}) & \lambda_{c} & \lambda_{vh} & \lambda_{vl} & 0 & 0 & 0 & 0\\ \mu_{c} & -(\mu_{c}+\lambda_{vh}+\lambda_{vl}) & 0 & 0 & \lambda_{vh} & \lambda_{vl} & 0 & 0\\ \mu_{vh} & 0 & -(\mu_{vh}+\lambda_{c}+\lambda_{vl}) & 0 & \lambda_{c} & 0 & \lambda_{vl} & 0\\ \mu_{vl} & 0 & 0 & -(\mu_{vl}+\lambda_{c}+\lambda_{vh}) & 0 & \lambda_{c} & \lambda_{vh} & 0\\ 0 & \mu_{vh} & \mu_{c} & 0 & -(\mu_{c}+\mu_{vh}+\lambda_{vl}) & 0 & 0 & \lambda_{vl}\\ 0 & \mu_{vl} & 0 & \mu_{c} & 0 & -(\mu_{c}+\mu_{vl}+\lambda_{vh}) & 0 & \lambda_{vh}\\ 0 & 0 & \mu_{vl} & \mu_{vh} & 0 & 0 & -(\mu_{vh}+\mu_{vl}+\lambda_{c}) & \lambda_{c}\\ 0 & 0 & 0 & 0 & 0 & \mu_{vh} & \mu_{c} & -(\mu_{c}+\mu_{vh}) \end{array}\right] \end{array}$$

The flow balance equations in 18 represent the system of balance equations that define the equilibrium conditions between state transitions, ensuring a stable probability distribution over time.

(18) $$\begin{array}{cc} \pi_{I}\left(\lambda_{c}+\lambda_{vh}+\lambda_{vl}\right) & =\pi_{C}\mu_{c}+\pi_{V_{h}}\mu_{vh}+\pi_{V_{l}}\mu_{vl},\\ \pi_{C}\left(\mu_{c}+\lambda_{vh}+\lambda_{vl}\right) & =\pi_{I}\lambda_{c}+\pi_{C_{vh}}\mu_{vh}+\pi_{C_{vl}}\mu_{vl},\\ \pi_{V_{h}}\left(\mu_{vh}+\lambda_{c}+\lambda_{vl}\right) & =\pi_{I}\lambda_{vh}+\pi_{V_{h}V_{l}}\mu_{vl}+\pi_{C_{vh}\mu_{c}},\\ \pi_{V_{l}}\left(\mu_{vl}+\lambda_{vh}+\lambda_{c}\right) & =\pi_{I}\lambda_{vl}+\pi_{C_{vl}}\mu_{c}+\pi_{V_{h}V_{l}}\mu_{vh},\\ \pi_{C_{vh}}\left(\mu_{vh}+\mu_{c}+\lambda_{vl}\right) & =\pi_{V_{h}}\lambda_{c}+\pi_{C}\lambda_{vh}\\ \pi_{C_{vl}}\left(\mu_{c}+\mu_{vl}+\lambda_{vh}\right) & =\pi_{V_{l}}\lambda_{c}+\pi_{C}\lambda_{vl}+\pi_{CVh_{vl}}\mu_{vh},\\ \pi_{V_{h}V_{l}}\left(\mu_{vh}+\mu_{vl}+\lambda_{c}\right) & =\pi_{V_{h}}\lambda_{vl}+\pi_{V_{l}}\lambda_{vh}+\pi_{{CVh}_{vl}}\mu_{c}\\ \pi_{{CVh}_{vl}}\left(\mu_{c}+\mu_{vl}\right) & =\pi_{C_{vh}}\lambda_{vl}+\pi_{C_{vl}}\lambda_{vh}+\pi_{V_{h}V_{l}}+\lambda_{c}. \end{array}$$

Equation (19) provides the normalization constraint, which guarantees that the sum of all state probabilities equals one, maintaining the consistency of the Markov model.

(19) $$\pi_{I}+\pi_{C}+\pi_{V_{h}}+\pi_{V_{l}}+\pi_{C_{vh}}+\pi_{C_{vl}}+\pi_{V_{h}V_{l}}+\pi_{{CVh}_{vl}}=1,$$

**Figure 9:**
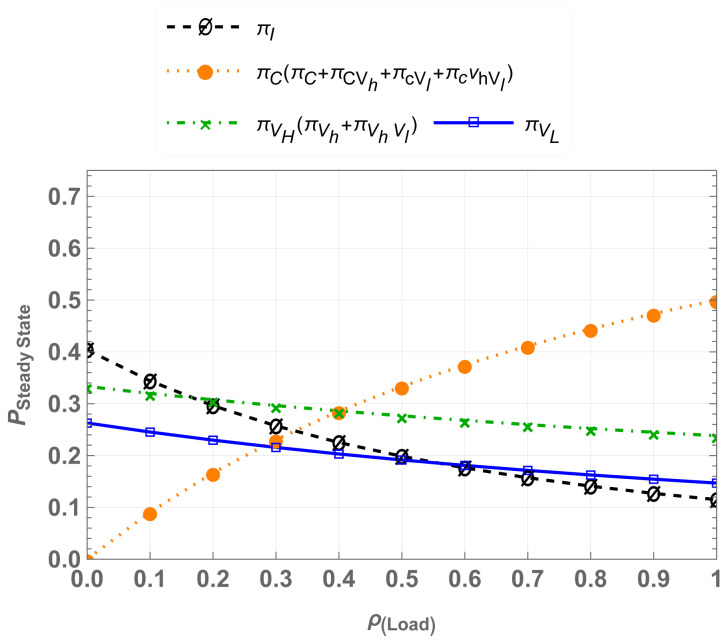
Steady-state probabilities for CU-VT-AMSA.

The steady-state probabilities $\pi_{I},\pi_{C},\pi_{V_{h}},\pi_{V_{l}},\pi_{C_{vh}},\pi_{C_{vl}},\pi_{V_{h}V_{l}},\mathrm{and}\pi_{{CVh}_{vl}}$ are obtained by solving these equations. For varying traffic loads $\rho =\frac{\lambda_{c}}{\mu_{c}}$, the steady-state probabilities of CU-VT-AMSA are shown in Figure 9. The results demonstrate that improved Spectrum Utilization and $V_{lp}$ reduced drop rates with the help of waiting mechanisms, ensuring minimal idle spectrum time, which results in higher Throughput achieved through coexistence and efficient resource allocation, and QoS Maintenance, which guarantees service for delay-sensitive $V_{hp}$ traffic. We will evaluate the performance of the proposed strategy in detail in the next section.

## 5. Simulation and Results

This section evaluates the proposed CU-VT-AMSA strategy performance using steady-state analysis and compares it with baseline schemes: CU-VT [9], CU-VT-IW [9,20], and CU-VT-HB [9,22,23]. The evaluation parameters include Throughput, Average Delay, Spectrum Utilization, and Steady-State Probabilities. The evaluation parameters used for the evaluation of these schemes are as described below. The simulation assumes a single cellular channel $N_{ch}$ = 1, with a load on the network ρ ranging from 0.0 to 1.0, capturing varying traffic intensities. CUs arrive at an average rate of $\lambda_{c}$ = 4 and depart at $\mu_{c}$ = 4. High-priority vehicular users $V_{hp}$ have a mean arrival rate of $\lambda_{hp}$ = 1 and departure rate of $\mu_{hp}$ = 2, while low-priority vehicular users $V_{lp}$ also arrive at $\lambda_{lp}$ = 1 and depart at $\mu_{lp}$ = 2. The system uses 5G data rates, with an interweave transmission rate $R_{i}$ = 1 Gbps and an underlay transmission rate $R_{u}$ = 300 Mbps when operating under low-power conditions. The channel bandwidth *W* is set to 100 MHz, consistent with 5G spectrum allocation. Each user’s average file size is assumed to be 5 MB, representing typical vehicular IoT data transmission demands. These parameters provide a robust foundation for analyzing the performance of the proposed spectrum access mechanisms in dynamic vehicular communication environments [9,24,25].

### 5.1. Steady-State Probability

**Figure 10:**
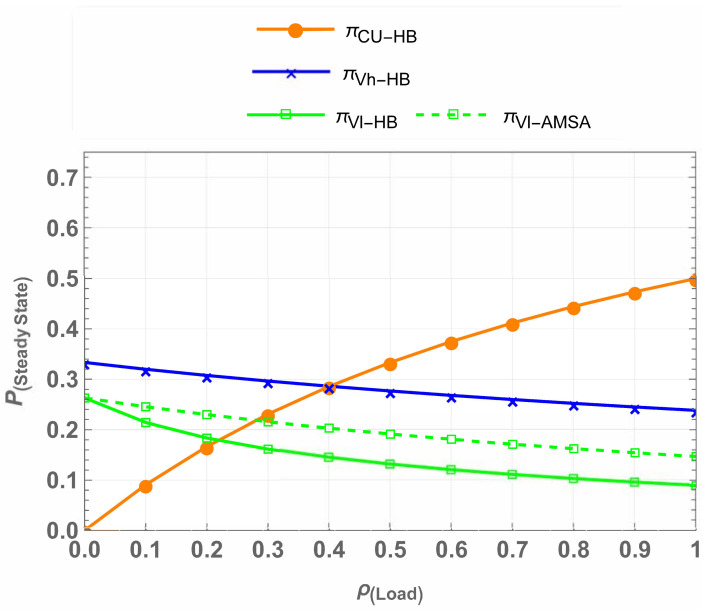
Comparison of steady-state probabilities across schemes.

The steady-state probabilities illustrate how often the system occupies each state and enable quantitative evaluation of system performance. Figure 10 shows the steady-state probabilities for $\pi_{CU}$, $\pi_{Vh}$, and $\pi_{Vl}$ in the IW, HB, and AMSA schemes. There are three main points to note here. First, the $\pi_{CU}$ occupies the spectrum uninterrupted in all cases, ensuring the spectrum is always available for CU, the highest-priority user in the network having critical data to transmit. This guarantees the safety and reliability of CU communication. Second, $\pi_{Vh}$ and $\pi_{Vl}$ opportunistically access the spectrum. For $\pi_{Vh}$, all three cases perform the same, which means the introduction of the waiting state does not compromise on $V_{hp}$. Lastly, in the $\pi_{Vl}$ case, the steady-state probabilities in AMSA achieve superior performance compared to IW and HB, showcasing improved resource allocation and spectrum utilization.

**Figure 11:**
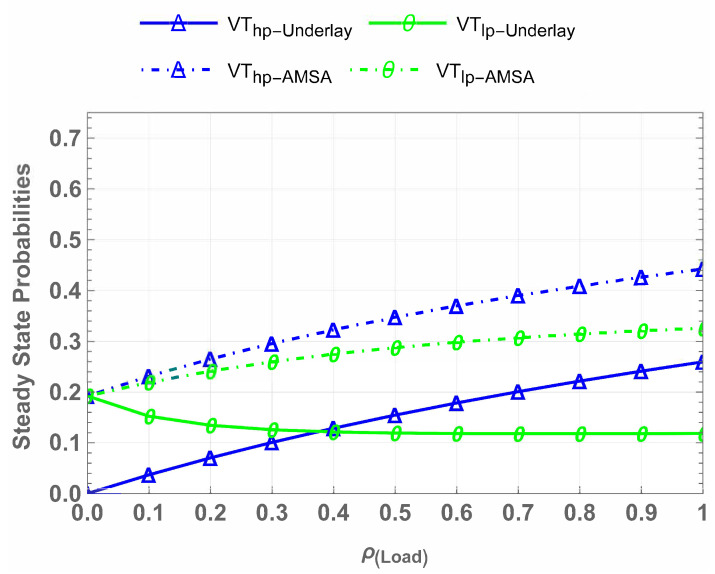
Impact of waiting, underlay, and AMSA mechanisms on $VT_{hp}$ and $VT_{lp}$.

Figure 11 highlights the impact of waiting and underlay mechanisms in CU-VT-AMSA. The steady-state probabilities for $\pi_{Vh}$ and $\pi_{Vl}$ improve significantly due to the introduction of waiting states, even under higher traffic loads. The AMSA ensures efficient spectrum utilization for $\pi_{Vh}$ and $\pi_{Vl}$ users while maintaining QoS for CUs.

### 5.2. Spectrum Utilization

**Figure 12:**
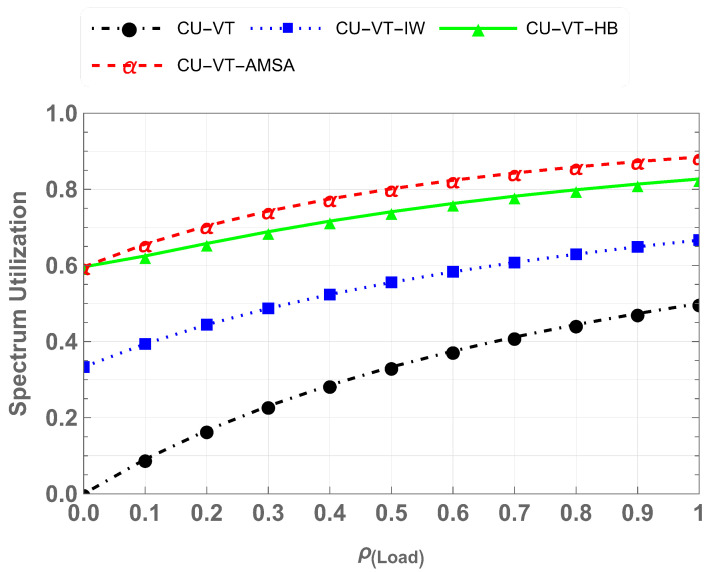
Comparison of spectrum utilization across schemes.

Spectrum utilization is measured as the ratio between the amount of time the spectrum is utilized by CU, $VT_{hp}$, and $VT_{lp}$ versus the total time. Figure 12 shows a comparison of spectrum utilization across all the scenarios developed in this work: CU-VT, CU-VT-IW, CU-VT-HB, and CU-VT-AMSA. Spectrum utilization measures the ratio of active spectrum usage time to the total time, and it increases with the number of active users in the system. The CU-VT scheme demonstrates minimal utilization, around 0.5, as it only accommodates CUs. In contrast, the CU-VT-AMSA scheme achieves maximum utilization, around 0.89, by enabling coexistence and waiting mechanisms, which allow more users to opportunistically access the spectrum without disrupting CU communication. The results demonstrate that at maximum load, CU-VT-AMSA performs 56% better than CU-VT, 29% better than CU-VT-IW, and 8% better than CU-VT-HB when compared for spectrum utilization.

**Figure 13:**
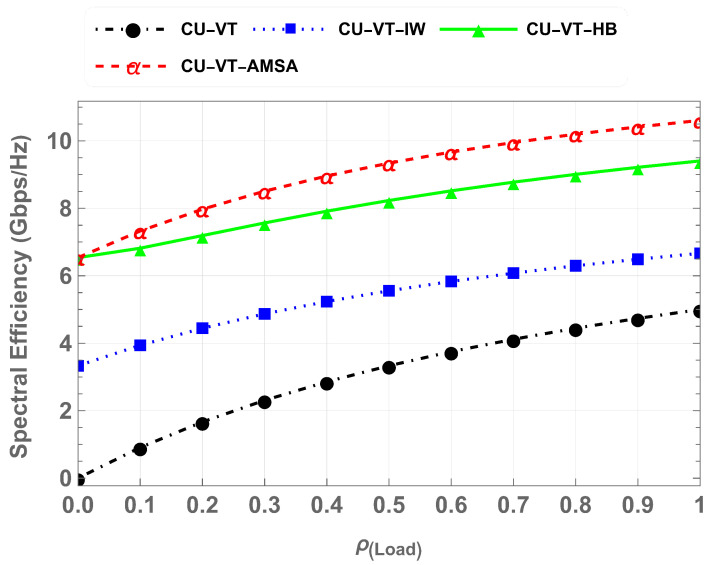
Comparison of spectrum efficiency across schemes.

Figure 13 illustrates the spectrum efficiency measured in Gbps/Hz for varying network loads across the same schemes. Even at the highest load, the CU-VT-AMSA scheme achieves a spectrum efficiency of around 10.5 Gbps/Hz, outperforming CU-VT, which is around 5 Gbps/Hz, CU-VT-IW, which is around 6.8 Gbps/Hz, and CU-VT-HB, which is around 9.3 Gbps/Hz. The results demonstrate that at maximum load, there is a 12.1% performance difference between CU-VT-AMSA and CU-VT-HB. Similarly, there is a 42.7% and 70.9% performance difference between CU-VT-AMSA compared to CU-VT-IW and CU-VT. The results demonstrate the effectiveness of AMSA in achieving optimal resource allocation and utilization under dynamic vehicular IoT conditions.

### 5.3. Throughput

**Figure 14:**
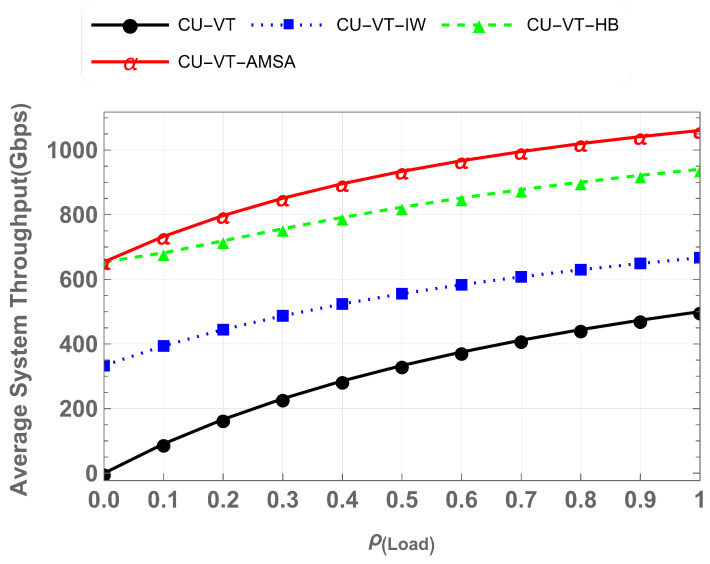
Average throughput comparison for all cases.

The throughput represents the total data successfully transmitted in Gbps under varying network loads, reflecting the effectiveness of the spectrum utilization mechanisms. Figure 14 compares the average throughput achieved by CU-VT, CU-VT-IW, CU-VT-HB, and CU-VT-AMSA for all users. At maximum load, CU-VT-AMSA achieves the highest throughput of 1050 Gbps, outperforming CU-VT-HB (930 Gbps), CU-VT-IW (660 Gbps), and CU-VT (500 Gbps). This translates to an approximate 13% improvement over CU-VT-HB, a 59% improvement over CU-VT-IW, and a remarkable 110% improvement over CU-VT. These results demonstrate the superior throughput of AMSA by managing spectrum resources and accommodating diverse traffic.

**Figure 15:**
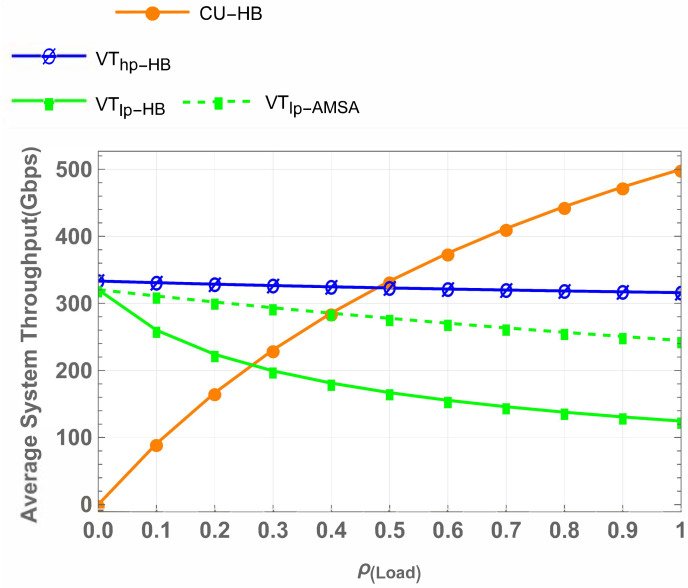
Detailed throughput comparison for specific user categories.

Figure 15 provides a detailed breakdown of throughput performance for specific user categories. For CU at full load, the throughput remains constant at 500 Gbps across all schemes, highlighting that AMSA does not compromise the CU communication. High-priority vehicular traffic ($V_{hp}$) also maintains a steady throughput of 320 Gbps in all schemes, demonstrating consistent QoS provisioning for delay-sensitive applications. In contrast, low-priority vehicular traffic ($V_{lp}$) exhibits significant variation. While CU-VT-IW and CU-VT-HB achieve a throughput of 120 Gbps for $V_{lp}$, CU-VT-AMSA doubles this performance to 240 Gbps. This 100% improvement reflects the effectiveness of AMSA’s waiting mechanisms and hybrid access strategies in accommodating delay-tolerant traffic.

### 5.4. Extended Data Delivery Time

**Figure 16:**
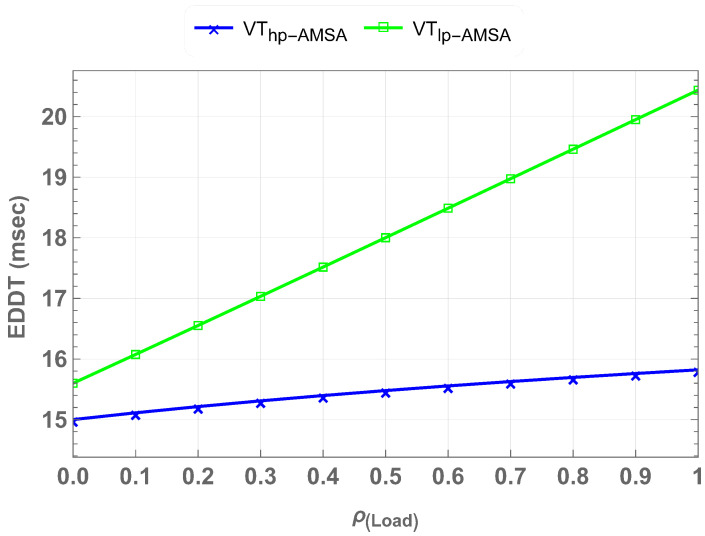
Comparison of EDDT for high-priority and low-priority traffic under AMSA at half and full loads, highlighting the scheme’s effectiveness in reducing delays for low-priority users.

The Extended Data Delivery Time (EDDT) represents the total time required to successfully deliver data packets under varying network loads. This reflects the efficient delay management strategy of AMSA in the V-IoT system. EDDT shows the system’s ability to manage packet delivery delays, which is critical for vehicular applications to maintain seamless user experiences. Figure 16 compares the EDDT for high-priority ($V_{hp}$) and low-priority ($V_{lp}$) traffic under the AMSA scheme at varying loads. At full load, $V_{hp}$-AMSA achieves an EDDT of 15.8 ms, while $V_{lp}$-AMSA records an EDDT of 20.4 ms. There is an approximately 23% difference in these values. At half load, the EDDT values for $V_{hp}$-AMSA and $V_{lp}$-AMSA reduce to 15.4 ms and 18 ms, which is approximately a 15% difference between both values. These results highlight the ability of the AMSA scheme to maintain low latency for high-priority traffic while effectively managing delays for low-priority traffic at varying load conditions.

**Figure 17:**
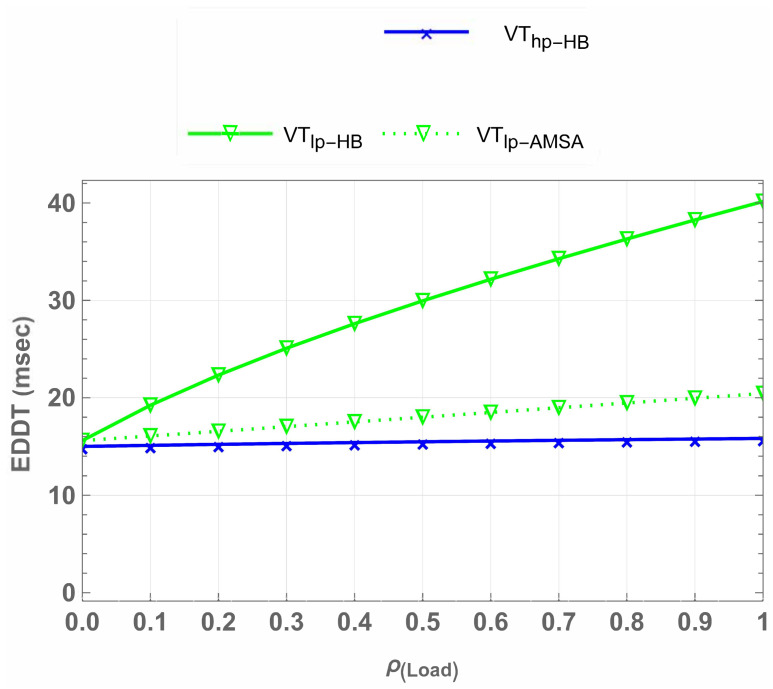
EDDT comparison across spectrum access schemes, showing AMSA’s superior performance for low-priority users at both half and full loads.

Figure 17 further explores the EDDT for high-priority and low-priority vehicular traffic across different spectrum access schemes, including IW, HB, and AMSA. For $V_{hp}$, the EDDT remains consistent across all schemes, at approximately 15 ms, regardless of the network load. This consistency ensures uninterrupted service for delay-sensitive high-priority traffic, which is essential for high-priority traffic in vehicular networks. However, $V_{lp}$ experiences significant improvements under AMSA. At full load, $V_{lp}$-AMSA reduces EDDT to 21 ms, compared to 40 ms for IW and HB, representing a 47.5% decrease in EDDT. Similarly, at half load, $V_{lp}$-AMSA achieves an EDDT of 17 ms, outperforming IW and HB, which remain at 30 ms, resulting in a 55.3% decrease in EDDT. This demonstrates the effectiveness of AMSA in minimizing delivery delays for low-priority traffic while ensuring reliable service for high-priority users.

The waiting mechanism under AMSA reduces contention for spectrum resources among low-priority users. These findings confirm AMSA’s superior delay management capabilities, particularly for low-priority traffic. AMSA’s optimization of spectrum usage and equitable service allocation make it a robust solution for a dynamic V-IoT environment.

### 5.5. Computational Complexity Analysis

To evaluate the scalability and real-time feasibility of AMSA, we analyze its computational complexity and compare it with existing models, including CU-VT-HB and CU-VT-IW. The complexity is assessed based on two key aspects, the CTMC steady-state computation and the adaptive spectrum mode selection process. Each model requires solving the steady-state probability distribution of the CTMC, governed by the balance equation:

(20) $$Q\cdot ß=0,\;\sum_{i=1}^{S}\pi_{i}=1$$

where Q is the transition rate matrix, and ß is the steady-state probability vector. The number of states, *S*, depends on the number of vehicles, $N_{v}$, and their access modes.

AMSA incorporates dynamic mode selection, incorporating interweave, underlay, and coexistence, leading to a state complexity of $O(N_{v}^{2})$. The matrix inversion required for solving the steady-state probabilities has a worst-case complexity of $O(S^{3})$, but using the iterative method, the complexity is reduced to $O(S^{2})$. The CU-VT-HB, which employs hybrid access, also results in a state complexity of $O(N_{v}^{2})$ but CU-VT-IW, which only considers interweave access, has a reduced state complexity of $O(N_{v})$, making it computationally less expensive.

The decision-making process in AMSA dynamically selects between different spectrum access modes. The computational steps include checking idle channel availability (O(1)), determining priority among CU, $VT_{hp}$, and $VT_{lp}$ (O(1)), and adjusting the spectrum access mode accordingly (O(1)). Since each decision step requires only constant-time operations, the total decision-making complexity for AMSA remains O(1). CU-VT-HB and CU-VT-IW, which rely on predefined priority rules without additional computations, have a similar decision-making complexity. The overall computational complexity of AMSA compared with CU-VT-HB and CU-VT-IW is summarized in Table 1.

AMSA provides dynamic adaptability while maintaining computational efficiency. Unlike CU-VT-IW, which has lower complexity but lacks underlay access, AMSA ensures better spectrum utilization while keeping decision-making operations at O(1). Simulation results confirm that AMSA maintains real-time feasibility with an average decision-making delay of less than 1 ms, making it suitable for large-scale vehicular networks.

## 6. Conclusions

This paper presented an AMSA scheme designed to enhance the QoS in V-IoT systems. By integrating coexistence mechanisms, waiting states, and hybrid spectrum access strategies, AMSA addresses key challenges, such as spectrum under-utilization and inconsistent QoS for multi-class traffic. The steady-state analysis using CTMC modeling demonstrates AMSA’s superiority over traditional and hybrid spectrum access schemes. Key performance metrics, including throughput, spectrum utilization, spectrum efficiency, and EDDT, were evaluated to validate AMSA’s effectiveness. The results showed significant improvements, with CU-VT-AMSA achieving a 110% higher throughput than CU-VT and a 59% enhancement over CU-VT-IW. AMSA also reduced EDDT for low-priority traffic by up to 47.5% compared to IW and HB schemes while ensuring uninterrupted service for critical and high-priority users.

Aside from its current use, AMSA also promises future potential by leveraging machine learning techniques. Future research can explore reinforcement learning-based spectrum access techniques that enable real-time intelligent decision-making based on network status and traffic demand patterns. Federated learning techniques could be employed to allow vehicles to collaboratively optimize spectrum access decisions without sacrificing data privacy. AMSA can be augmented with AI-based predictive modeling to make it more responsive in highly dynamic vehicular environments, enabling proactive spectrum allocation based on anticipated congestion levels and user mobility patterns. The findings in this study bring to light AMSA as a promising scalable and efficient spectrum access solution for future vehicular networks. Its flexibility and efficiency make it an ideal candidate for incorporating into intelligent transportation systems and future 6G-enabled vehicular communication networks.

## Figures and Tables

**Table 1:** Computational complexity comparison.

Technique	CTMC State Complexity	Decision Complexity	Total Complexity
AMSA (Proposed)	$O(N_{v}^{2})$	O(1)	$O(N_{v}^{2})$
CU-VT-HB	$O(N_{v}^{2})$	O(1)	$O(N_{v}^{2})$
CU-VT-IW	$O(N_{v})$	O(1)	$O(N_{v})$

## Data Availability

Dataset available on request from the authors.
